# Reforming the Materia Medica

**Published:** 1893-10

**Authors:** 


### HOMŒOPATHIC PHYSICIAN,

A MONTHLY JOURNAL OF

HOMŒOPATHIC MATERIA MEDICA AND CLINICAL MEDICINE.

"If our school ever gives up the strict inductive method of Hahnemann, we are lost, and deserve only to be mentioned as a caricature in the history of medicine."—CONSTANTINE HERING.

Vol. XIII. OCTOBER, 1893. No. 10.

### EDITORIAL.

REFORMING THE MATERIA MEDICA.—It is so much more easy to pull down than to build up; it is so much more easy to point out the defects of a picture than it is to paint it; it is so much more easy to discredit a literary production than to create one that critics are much more numerous than artists. The critic pulls down; he is essentially a destroyer.

The unreasoning forces of nature—fire, flood, and storm—are destroyers. They devastate in an hour beauties of landscape that took ages for their growth.

The predatory animals of the wilderness are destroyers also. They exterminate in a few minutes other creatures that took years to attain their growth.

Thus the whole tendency of nature is toward destruction—the pulling down of what was slowly and maybe painfully built up.

Man, on the other hand, in the pride of his intellect and the triumph of his civilization, considers it his special mission to create, to discover, to invent—in a word, to build up. His creations attain perfection slowly and painfully, but, nevertheless, surely. Architectural grandeur developed from the tent. That triumphant annihilator of space, the locomotive engine, developed from incredibly crude conceptions.

The curious inquirer may learn the truth of these two statements, if he visit the great Chicago Fair, where, in the Anthropological Building, he will see the examples of the wondrous advances in architecture; or in the Transportation Building, where he will be able to trace the development of the present magnificent models of the locomotive engine from the first rude constructions.

Because these early attempts failed of the duty exacted from them, they were not, therefore, abandoned and the idea declared absurd. On the contrary, the defects were attentively studied and slowly, painfully, and, it may be, awkwardly, they were remedied one after another until success was assured.

We homœopathists appear to be different in this respect. We have had bequeathed to us by the old painstaking, faithful masters a materia medica pura. We find it imperfect and charged with errors—or what we, in our intellectual arrogance, conceive to be errors, inconsistencies, and absurdities. We wish to eliminate these errors and absurdities. How? By faithful trial and careful observation upon the sick? No! We would sift these errors with a rational sieve; or, we would pull down the entire structure and, setting the ruin behind us, abandon the whole principle and enter another and a Philistine temple, there to offer our devotions to their idols. Thus we identify ourselves in our mode of thought with the blind forces of nature whose principal work is to destroy. There is no high order of intellectuality in such a proceeding. There is no element of progress in it. There can be nothing gained by it. It is not the method followed by other scientific laborers in other fields. *They* do not throw away all the fabric they have created. *They* do not destroy the steps by which they hoped to ascend. Then we must be wrong.

If we throw away what we have, what shall we do for a substitute? We have no resource, and must betake ourselves into the allopathic school, and that is a confession that Homœopathy is a delusion.

There are a few who will not follow us. Withdrawing to a camp by themselves, they hold their materia medica with a careful and a greedy clasp, and, turning their backs upon our beckonings, they eagerly scan the symptomatology for the simillimum which their benighted minds tell them will, if found, surely enable them to heal the sick.

It is they who will not abandon the keynote—the sweating during the sleep of Calcarea and Silicea; the flushes of heat of Sulphur; the sensitiveness of the neck to the light pressure of the collar of Lachesis, and the hundreds of like indications with which they crowd their minds to the exclusion of the germ theory, and all the details of modern pathology.

They report their triumphs in the journals. But as they are not developed along the lines of modern scientific pathology, we despise them and ignore them.

It is this handful of men who will not suffer the temple of materia medica to be torn down, but are holding it intact for the appreciation and use of a later and, perhaps, a wiser generation.

